# Genetically predicted anti‑rubella virus IgG levels and dermatitis risk: A two‑sample Mendelian randomization study

**DOI:** 10.1016/j.virusres.2026.199726

**Published:** 2026-04-15

**Authors:** Zidan Ouyang, Jiajian Lin, Jing Wu, Xiaojun Wang, Haijiao Wang, Li Chen

**Affiliations:** a Guangzhou University of Chinese Medicine, Guangzhou 510006, China; b The First Affiliated Hospital of Guangzhou University of Chinese Medicine, Guangzhou 510405, China

**Keywords:** Dermatitis, IgG, Mendelian randomization, Allergic contact dermatitis, Atopic dermatitis

## Abstract

**Background:**

Dermatitis is a common inflammatory skin condition with multifactorial origins. Although immune responses following vaccination or natural infection have been linked to dermatological outcomes, the causal significance of virus-specific immune markers remains unclear. This study used Mendelian randomization (MR) to investigate whether genetically predicted virus-specific IgG levels are associated with dermatitis risk.

**Methods:**

A two‑sample MR analysis was performed using genome‑wide association study (GWAS) summary statistics for IgG levels against eight common viral antigens from the Milieu Intérieur cohort and dermatitis outcomes (allergic contact dermatitis, atopic dermatitis, and other contact dermatitis) from large European cohorts. The inverse variance weighted method was used as the primary analysis method, complemented by MR‑Egger, weighted median, weighted mode, MR‑PRESSO, and leave‑one‑out sensitivity analyses.

**Results:**

Genetically predicted anti-rubella virus IgG levels showed a suggestive association with a lower risk of allergic contact dermatitis (OR = 0.60, 95% CI: 0.41–0.88, P = 0.009). This association did not survive Bonferroni correction and should be interpreted cautiously as such. No significant associations were observed for other virus-specific IgG levels or dermatitis outcomes. Sensitivity analyses indicated no evidence of horizontal pleiotropy or influence from any single genetic variant.

**Conclusion:**

This MR study provides suggestive evidence that genetically predicted anti-rubella IgG levels may be associated with reduced allergic contact dermatitis risk. However, the findings are exploratory, do not distinguish between vaccine-induced and infection-induced immunity, and require replication and mechanistic validation. No causal associations were found for other virus-specific IgG traits.

## Highlights

• Suggests anti-rubella IgG may reduce ACD risk (OR 0.60; P = 0.009).
• No causal links found for other vaccines and dermatitis outcomes.
• MR-Egger shows no strong pleiotropy; MR-PRESSO corrected an outlier.
• 47 IVs identified for vaccination exposure in two-sample MR design.
• Findings point to vaccine-specific, not universal, dermatological effects.

## 1 Introduction

Dermatitis refers a group of inflammatory skin disorders with diverse etiologies. Among these, atopic dermatitis (AD) and allergic contact dermatitis (ACD) are two major subtypes with considerable public health relevance. AD affects up to 20% of children and 10% of adults, while ACD has a prevalence of approximately 20% in the general population (Langan et al., 2020; Nassau and Fonacier, 2020). The development of these conditions is multifactorial, driven by genetic predisposition, environmental exposures, immune dysregulation, and lifestyle factors (Langan et al., 2020; Olusegun and Martincigh, 2021). Identifying upstream immunological determinants that may influence dermatitis susceptibility is therefore essential for improving prevention and management strategies.

Immune responses to viral antigens, whether from natural infection or vaccination, have long been hypothesized to influence allergic and inflammatory pathways. Epidemiological studies examining the link between childhood vaccinations and eczema-related outcomes have yielded inconsistent findings. Some investigations found no increased risk of allergic disease following routine immunizations and even suggested potential reductions in eczema severity (Gruber, Warner, Hill, Bauchau, and Group, 2008). Other reports described increased AD incidence following measles infection or measles‑mumps‑rubella vaccination (Olesen, Juul, and Thestrup-Pedersen, 2003). A recent meta‑analysis similarly found no overall increase in allergic disease risk associated with childhood vaccines, while pointing to potential protective associations for certain vaccines such as BCG and measles (Navaratna et al., 2021). These heterogeneous findings highlight the complexity of immune–skin interactions and underscore the need for approaches that can better disentangle correlation from causation.

Examining immunoglobulin profiles may provide insight into these relationships. IgM typically reflects acute infection, IgG represents long‑term immune memory, and IgE plays a central role in allergic sensitization and type I hypersensitivity reactions (Jones, Savulescu, Brombacher, and Hadebe, 2020; Knol and van Neerven, 2024). Virus‑specific IgG levels, in particular, capture cumulative immune exposure from both vaccination and natural infection. Notably, genetically determined variation in IgG levels may influence downstream immune responses independent of environmental exposures. However, whether such genetically predicted differences in IgG contribute to dermatitis susceptibility remains unclear.

Mendelian randomization (MR) offers a powerful framework for investigating potential causal relationships by using genetic variants as instrumental variables (IVs). Because genetic variants are randomly allocated at conception, MR can help reduce confounding and reverse causation, two key limitations of observational studies (Birney, 2022; Ference, Holmes, and Smith, 2021). Despite its advantages, MR has seldom been used to explore the immunological determinants of dermatitis, and no prior study has systematically evaluated whether genetically predicted virus‑specific IgG levels influence the risk of AD or ACD.

The present study was designed to address this gap by conducting a two‑sample MR analysis to examine the potential causal associations between genetically predicted IgG levels against eight common viral antigens and multiple dermatitis outcomes. By leveraging large‑scale genome‑wide association study (GWAS) datasets, we aim to provide genetically informed evidence on whether long‑term antiviral immune memory may contribute to dermatitis risk. This work may help clarify the immunological pathways underlying dermatitis and generate hypotheses for future mechanistic research.

## 2 Material and methods

### 2.1 Study design

**Fig. 1:**
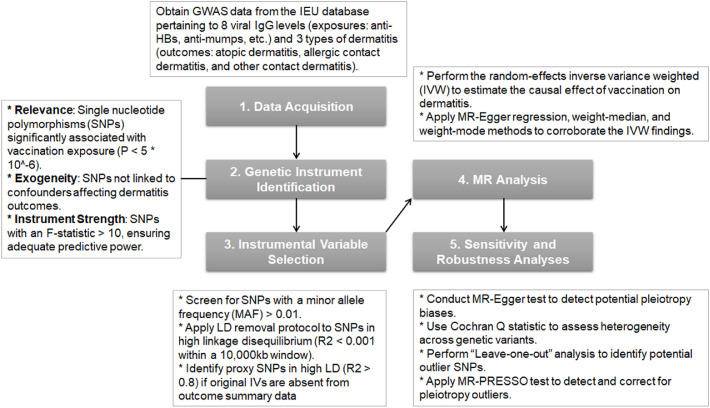
**Study Design and Flow Charting.** A schematic overview of the two‑sample MR framework used to evaluate the potential causal effects of genetically predicted virus‑specific IgG levels on dermatitis outcomes.

This MR study was conducted in accordance with the STROBE-MR guidelines (Skrivankova et al., 2021). It was designed to investigate the potential causal relationship between virus‑specific IgG levels and the development of dermatitis using a two‑sample MR approach. Ethical approval and informed consent have been described in the original GWAS publications. A schematic overview of the study design is presented in Fig. 1. The analysis adhered to the three core assumptions of MR: a. genetic variants are robustly associated with the exposure; b. genetic variants are independent of confounding variables; c. genetic variants influence the outcome solely through the exposure and not via alternative pathways (Davies, Holmes, and Davey Smith, 2018).

### 2.2 Data source

Virus‑specific IgG levels were treated as the exposure, whereas dermatitis, including allergic contact dermatitis (ACD), atopic dermatitis (AD), and other contact dermatitis (OCD), were considered as the outcomes. The genome-wide association studies (GWAS) summary dataset for AD was obtained from a meta-analysis of FinnGen, the Estonian Biobank, and the UK Biobank, totalling AD 796,661 samples in the study(Sliz et al., 2022). The definition of AD is based on ICD-10 code L20.0, ICD-8 code 69,100, or ICD-9 code 6918B The GWAS data for ACD (200,944 samples) and OCD (200,043 samples) were obtained from FINNGEN R5. ACD is registered as ICD-10 code L23 or ICD-9 code 6920B/6921B/6922B/6924B/6924D/6924H/6924L/6925B/6925D/6926B/6926D, while OCD is defined by ICD-10 code L25/L24, ICD-9 code 692, or ICD-8 code 69281.

Summary-level data for the exposures, including IgG levels against eight common viral antigens, were obtained from a large GWAS of the Milieu Intérieur cohort (Scepanovic et al., 2018). This well-characterized cohort consists of 1000 healthy individuals of French descent, stratified by age (from 20 to 69 years) and sex. The study measured IgG levels for antigens related to hepatitis B virus (anti-HBs, anti-HBc), measles virus, mumps virus, rubella virus, varicella zoster virus, herpes simplex virus 1 & 2 (HSV-1, HSV-2), and influenza A virus. These IgG levels reflect immune responses generated from both childhood vaccinations (e.g., measles, mumps, rubella) and natural infections. The original study employed a range of clinical-grade serological assays to quantify these IgG levels, details of which are described in Scepanovic et al. (Scepanovic et al., 2018).

To avoid misinterpretation, we emphasize that virus‑specific IgG levels represent cumulative immune memory from both vaccination and natural infection and should not be interpreted as direct proxies for vaccination exposure.

The exposure and outcome GWAS datasets were derived from independent cohorts with no sample overlap, minimizing bias due to correlated errors. To reduce population stratification, all analyses were restricted to individuals of European ancestry.

A complete list of the outcome traits and the 8 IgG levels, along with their respective aliases, GWAS IDs, sample sizes, and number of single nucleotide polymorphisms (SNPs), is presented in Table 1.

**Table 1:** The GWAS data for exposure and outcomes.

Trait	Diseases or Exposure	GWAS ID	Sample Size (case/control)	SNPs
Atopic dermatitis	Atopic dermatitis	ebi-a-GCST90027161	22,474/774,187	16,121,213
Allergic contact dermatitis	Allergic contact dermatitis	finn-b-L12_ALLERGICCONTACT	2204/198,740	16,380,431
Other contact dermatitis	Other contact dermatitis	finn-b-L12_OTHERCONTACT	1303/198,740	16,380,425
Anti-hepatitis B virus surface antigen (HBs) IgG levels	Anti-hepatitis B virus surface antigen (HBs) IgG levels	ebi-a -GCST006354	508	5278,042
Anti-mumps virus IgG levels	Anti-mumps virus IgG levels	ebi-a -GCST006352	912	5278,042
Anti-measles virus IgG levels	Anti-measles virus IgG levels	ebi-a-GCST006351	885	5278,042
Anti-rubella virus IgG levels	Anti-rubella virus IgG levels	ebi-a-GCST006353	935	5667,532
Anti-varicella zoster virus IgG levels	Anti-varicella zoster virus IgG levels	ebi-a-GCST006348	931	5278,042
Anti-herpes simplex virus 1 IgG levels	Anti-herpes simplex virus 1 IgG levels	ebi-a-GCST006346	645	5278,042
Anti-herpes simplex virus 2 IgG levels	Anti-herpes simplex virus 2 IgG levels	ebi-a-GCST006347	208	5278,042
Anti-influenza A virus IgG levels	Anti-influenza A virus IgG levels	ebi-a-GCST006350	777	5278,042

### 2.3 Instrumental variable selection

The selection of IVs for this study followed a stringent set of criteria to ensure robustness and validity. Initially, we screened for SNPs at the conventional genome-wide significance threshold (P < 5 × 10⁻⁸), but this yielded too few instruments for a robust analysis. A relaxed threshold of P < 5 × 10⁻⁶ was therefore adopted, which is commonly used in MR studies of immune traits when genome‑wide significant SNPs are insufficient for analysis (Long, Tang, Zhou, Zhao, and Zhu, 2023).

To address the impact of linkage disequilibrium (LD), a stringent LD removal protocol was employed. Specifically, SNPs are filtered based on an R^2^ threshold of <0.001 within a window size of 10,000 kb, effectively eliminating SNPs in high LD (Genomes Project et al., 2010). Where the chosen IVs are absent from the outcome’s summary data, the study proactively identifies proxy SNPs in high LD (R^2^ > 0.8) with the original IVs to ensure a reliable representation of the genetic effect (Bell et al., 2021). To avoid weak instrument bias, the strength of each IV is rigorously evaluated. This is accomplished by calculating the F statistic for every SNP within the IV, utilizing the formula: F = R^2^ * (N-2) / (1-R^2^). R^2^ represents the proportion of the exposure variance explained by the SNP. A minimum F‑statistic threshold of 10 was applied to ensure adequate predictive power (Burgess and Thompson, 2011). Through this rigorous selection process, the IVs selected were confirmed to be strongly associated with the exposure and served as reliable indicators, thereby strengthening the validity of the MR analysis.

### 2.4 MR analysis

The causal association between exposure and outcome was assessed using MR analysis, with a primary focus on the inverse variance weighted (IVW) method (Burgess, Butterworth, and Thompson, 2013). This approach provides the odds ratio (OR) and its corresponding 95% confidence interval (CI), offering a weighted average of effect sizes across different SNPs, where the weights are determined by the inverse of the SNPs’ variance. The IVW method serves as a cornerstone for interpreting MR results, providing a balanced and comprehensive view of the genetic associations. To ensure the robustness of the findings, additional MR methods were incorporated, including the MR-Egger regression, which accounts for potential pleiotropic bias by estimating an intercept term (Bowden, Davey Smith, and Burgess, 2015). This method helps ensure that causal effect estimates remain accurate even when some of the genetic instruments may affect the outcome through multiple pathways. Furthermore, the weighted median (WM) method was employed, which operates under the assumption that at least half of the IVs are valid, thereby enhancing robustness against outliers and providing a more nuanced understanding of the exposure-outcome relationship (Bowden, Davey Smith, Haycock, and Burgess, 2016). All analyses were performed using the "TwoSampleMR" R package version 4.3.2 (Hemani et al., 2018). To enhance interpretability and transparency, visualization techniques were utilized, including scatter plots and sensitivity analysis plots.

Given the multiple hypotheses tested (8 exposures against 3 outcomes), Bonferroni correction was applied to adjust for multiple comparisons, with a corrected significance threshold set at *P*-value < 0.002 (0.05/24). Results with a nominal significance level of P < 0.05 were also reported for exploratory purposes.

### 2.5 Sensitivity analysis

Sensitivity analyses were conducted to assess heterogeneity within the MR framework and to evaluate the robustness of the findings. Cochran’s Q test was used to assess heterogeneity among the IVs, with a *P*‑value > 0.05 suggesting a uniform distribution of effect estimates and minimal impact on the reliability of the IVW method (Greco, Minelli, Sheehan, and Thompson, 2015). MR-Egger regression was performed to investigate horizontal pleiotropy, a situation in which genetic variants influence the outcome through pathways independent of the exposure (Bowden et al., 2015). A statistically non‑significant intercept in the MR‑Egger regression indicates a low likelihood of pleiotropic effects, thereby supporting the integrity of the MR analysis. To identify and correct for outliers, the MR pleiotropy residual sum and outlier (MR-PRESSO) was employed, with SNPs showing a *P*-value < 0.05 (Verbanck, Chen, Neale, and Do, 2018). Causal estimates were recalculated after removing these outliers to adjust for potential horizontal pleiotropy and enhance the precision of the findings. Additionally, leave-one-out analysis was implemented to test the stability of the results (Cheng, Dekkers, and Fernando, 2021). This involved the step-by-step exclusion of each IV to assess its individual impact on the overall effect estimate, thereby ensuring that the conclusions were not disproportionately influenced by any single genetic variant.

## 3 Results

### 3.1 Instrument variable selection

In this study, a total of 47 SNPs associated with virus‑specific IgG levels were selected as IVs. The F statistics of all identified SNPs were exceeded 10, indicating no evidence of weak instrument bias (Table S1). Details of the IV selection for the MR analysis of IgG levels on dermatitis are provided in the Table S2.

### 3.2 Causal effects of genetically predicted virus IgG levels on dermatitis

**Fig. 2:**
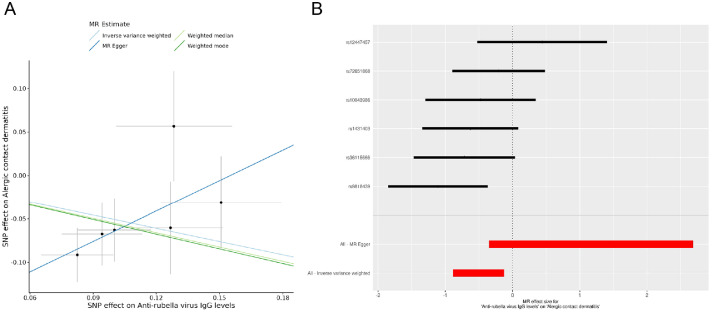
**The scatter and forest plot for the association between genetically predicted anti‑rubella virus IgG levels and allergic contact dermatitis (ACD).** (A) Scatter plot showing MR estimates of anti‑rubella virus IgG levels on ACD across different instrumental variables. (B) Forest plot displaying individual SNP effects and the overall MR estimate for anti‑rubella virus IgG levels on ACD.

The MR analysis provided insights into the potential causal relationship between genetically predicted virus-specific IgG levels and the risk of dermatitis. According to the IVW results, genetically predicted anti-rubella virus IgG levels showed a suggestive association with ACD (OR (95% CI): 0.6022 (0.4116 - 0.8811), *P* = 0.009), which was consistent with findings from the weighted median analysis (OR (95% CI): 0.5773 (0.3766 - 0.8851), *P* = 0.012) (Table 2). However, this association did not withstand Bonferroni correction (P > 0.002) and should therefore be interpreted with caution. The scatter and forest plots of positive results are shown in Fig. 2. No significant association were observed between other IgG levels and dermatitis using IVW methods, and the remaining three approaches (MR-Egger, WM, and weighted mode) yielded similar results (Table 2).

**Table 2:** Relationship between vaccination and dermatitis (positive result).

exposure	outcome	nsnp	method	or_ci	p
Anti-rubella virus IgG levels	Alergic contact dermatitis	6	Inverse variance weighted	0.6022 (0.4116 - 0.8811)	0.009
Anti-rubella virus IgG levels	Alergic contact dermatitis	6	MR Egger	3.2148 (0.7045 - 14.6694)	0.206
Anti-rubella virus IgG levels	Alergic contact dermatitis	6	Weighted median	0.5773 (0.3766 - 0.8851)	0.012
Anti-rubella virus IgG levels	Alergic contact dermatitis	6	Weighted mode	0.5693 (0.3191 - 1.0160)	0.115

### 3.3 Sensitivity analysis

MR-Egger regression analysis revealed no evidence of horizontal pleiotropy, and the results remained consistent across sensitivity analyses, as presented in Table 3. Cochran's Q test revealed substantial heterogeneity in the analyses of anti-rubella virus IgG levels and AD, anti-measles virus IgG levels and AD, as well as anti-rubella virus IgG levels and OCD (Table 3). MR-PRESSO identified outliers for anti-rubella virus IgG levels and AD (Table 4). After excluding these outliers and re‑running the analysis, no further outliers were detected (Table 4). Funnel plots revealed no evidence of horizontal pleiotropy (Fig. S1A), and leave-one-out sensitivity analysis demonstrated that no single SNP drove the overall estimate, supporting the stability of the findings. (Fig. S1B).

**Table 3:** Tests for horizontal pleiotropy and heterogeneity.

Exposure	Outcome	Heterogeneity		Pleiotropy	
		Q statistic (IVW)	*P* value	MR-Egger Intercept	*P* value
Anti-influenza A virus IgG levels	Atopic dermatitis	1.78	0.97	−2.55E-03	0.91
Anti-hepatitis B virus surface antigen (HBs) IgG levels	Atopic dermatitis	2.92	0.57	1.86E-02	0.49
Anti-mumps virus IgG levels	Atopic dermatitis	1.48	0.69	−1.57E-02	0.72
Anti-rubella virus IgG levels	Atopic dermatitis	13.41	0.02	6.09E-03	0.91
Anti-herpes simplex virus 2 IgG levels	Atopic dermatitis	3.13	0.87	2.23E-02	0.35
Anti-varicella zoster virus IgG levels	Atopic dermatitis	2.56	0.77	−7.42E-03	0.83
Anti-measles virus IgG levels	Atopic dermatitis	15.6	0.01	−6.29E-03	0.86
Anti-herpes simplex virus 1 IgG levels	Atopic dermatitis	0.39	0.82	−2.26E-02	0.74
Anti-influenza A virus IgG levels	Allergic contact dermatitis	5.56	0.59	6.35E-02	0.36
Anti-influenza A virus IgG levels	Other contact dermatitis	8.01	0.33	−8.56E-03	0.93
Anti-hepatitis B virus surface antigen (HBs) IgG levels	Allergic contact dermatitis	0.94	0.92	−7.15E-04	0.99
Anti-hepatitis B virus surface antigen (HBs) IgG levels	Other contact dermatitis	1.26	0.87	−1.98E-02	0.84
Anti-mumps virus IgG levels	Allergic contact dermatitis	0.60	0.90	−5.92E-02	0.64
Anti-mumps virus IgG levels	Other contact dermatitis	4.26	0.23	3.45E-02	0.88
Anti-rubella virus IgG levels	Allergic contact dermatitis	7.38	0.19	−1.81E-01	0.09
Anti-rubella virus IgG levels	Other contact dermatitis	13.38	0.02	1.12E-01	0.58
Anti-herpes simplex virus 2 IgG levels	Allergic contact dermatitis	5.03	0.66	3.97E-02	0.57
Anti-herpes simplex virus 2 IgG levels	Other contact dermatitis	6.26	0.51	1.28E-01	0.18
Anti-varicella zoster virus IgG levels	Allergic contact dermatitis	7.37	0.19	−1.23E-01	0.36
Anti-varicella zoster virus IgG levels	Other contact dermatitis	10.64	0.06	−1.19E-01	0.59
Anti-measles virus IgG levels	Allergic contact dermatitis	5.68	0.34	−1.17E-02	0.86
Anti-measles virus IgG levels	Other contact dermatitis	5.87	0.32	−5.77E-03	0.95
Anti-herpes simplex virus 1 IgG levels	Allergic contact dermatitis	1.93	0.59	−7.20E-02	0.56
Anti-herpes simplex virus 1 IgG levels	Other contact dermatitis	0.87	0.83	1.08E-02	0.94

**Table 4:** Results of MR-PRESSO.

Exposure	Outcome	Raw		Outlier corrected		Global *P*	Number of outliers	Distortion P
		OR (CI%)	*P*	OR (CI%)	*P*			
Anti-rubella virus IgG levels	Atopic dermatitis	0.91 (0.77 - 1.07)	0.31	0.99 (0.91 - 1.08)	0.81	0.047	rs1431403	<0.001
Anti-mumps virus IgG levels	Atopic dermatitis	0.85 (0.70 - 1.02)	0.17	NA (NA - NA)	NA	0.645		
Anti-herpes simplex virus 2 IgG levels	Atopic dermatitis	0.98 (0.96 - 1.01)	0.27	NA (NA - NA)	NA	0.895		
Anti-varicella zoster virus IgG levels	Atopic dermatitis	1.03 (0.90 - 1.18)	0.64	NA (NA - NA)	NA	0.779		
Anti-influenza A virus IgG levels	Atopic dermatitis	0.91 (0.83 - 1.00)	0.09	NA (NA - NA)	NA	0.969		
Anti-herpes simplex virus 1 IgG levels	Atopic dermatitis	0.87 (0.73 - 1.02)	0.19	NA (NA - NA)	NA	0.304		
Anti-measles virus IgG levels	Atopic dermatitis	1.05 (0.81 - 1.35)	0.75	1.04 (0.88 - 1.24)	0.66	0.018	rs718630\rs989134	1
Anti-hepatitis B virus surface antigen (HBs) IgG levels	Atopic dermatitis	0.99 (0.96 - 1.02)	0.45	NA (NA - NA)	NA	0.618		
Anti-rubella virus IgG levels	Allergic contact dermatitis	0.60 (0.41 - 0.88)	0.05			0.275		
Anti-mumps virus IgG levels	Allergic contact dermatitis	1.18 (0.73 - 1.92)	0.53			0.639		
Anti-herpes simplex virus 2 IgG levels	Allergic contact dermatitis	1.10 (0.80 - 1.50)	0.6			0.897		
Anti-varicella zoster virus IgG levels	Allergic contact dermatitis	1.03 (0.93 - 1.14)	0.62			0.641		
Anti-influenza A virus IgG levels	Allergic contact dermatitis	1.58 (0.79 - 3.18)	0.26			0.266		
Anti-herpes simplex virus 1 IgG levels	Allergic contact dermatitis	0.83 (0.58 - 1.19)	0.39			0.627		
Anti-measles virus IgG levels	Allergic contact dermatitis	0.97 (0.93 - 1.01)	0.24			0.934		
Anti-hepatitis B virus surface antigen (HBs) IgG levels	Allergic contact dermatitis	1.31 (0.82 - 2.09)	0.31			0.396		
Anti-rubella virus IgG levels	Other contact dermatitis	1.20 (0.56 - 2.55)	0.65			0.375		
Anti-mumps virus IgG levels	Other contact dermatitis	1.58 (0.53 - 4.68)	0.45			0.089		
Anti-herpes simplex virus 2 IgG levels	Other contact dermatitis	1.13 (0.58 - 2.20)	0.74	1.53 (0.91 - 2.57)	0.19	0.043	rs72651868	0.529
Anti-varicella zoster virus IgG levels	Other contact dermatitis	1.04 (0.98 - 1.12)	0.26			0.874		
Anti-influenza A virus IgG levels	Other contact dermatitis	0.65 (0.48 - 0.89)	0.08			0.843		
Anti-herpes simplex virus 1 IgG levels	Other contact dermatitis	1.03 (0.89 - 1.20)	0.68			0.553		
Anti-measles virus IgG levels	Other contact dermatitis	1.27 (0.44 - 3.72)	0.69			0.293		
Anti-hepatitis B virus surface antigen (HBs) IgG levels	Other contact dermatitis	1.11 (0.60 - 2.05)	0.76			0.383		

## 4 Discussion

This Mendelian randomization (MR) study investigated whether genetically predicted virus‑specific IgG levels influence the risk of dermatitis. Among the eight IgG traits examined, only anti‑rubella virus IgG levels demonstrated a suggestive association with allergic contact dermatitis (ACD). Although the direction and magnitude of the association were consistent across multiple MR methods, the result did not withstand Bonferroni correction and should therefore be interpreted with caution. Nevertheless, the consistency of evidence across sensitivity analyses, including MR‑Egger, weighted median, MR‑PRESSO, and leave‑one‑out approaches, suggests that the observed association is unlikely to be driven by horizontal pleiotropy or single‑variant effects.

A key consideration in interpreting these findings is the immunological role of IgG. Virus‑specific IgG reflects long‑term immune memory generated through both vaccination and natural infection. In contrast, IgM typically indicates acute infection, while IgE plays a central role in allergic sensitization and type I hypersensitivity reactions (Jones et al., 2020; Knol and van Neerven, 2024). The potential inverse association between genetically predicted anti‑rubella IgG levels and ACD risk may reflect broader immune regulatory mechanisms rather than a direct effect of vaccination. For example, IgG antibodies can modulate immune responses through Fcγ receptor signaling, immune complex clearance, and competitive inhibition of IgE‑mediated pathways (Hematianlarki and Nimmerjahn, 2024). These mechanisms may theoretically influence susceptibility to delayed‑type hypersensitivity reactions such as ACD. However, the present MR design cannot determine whether the observed association reflects immunological memory from vaccination, natural infection, or genetically driven differences in humoral immune responsiveness.

It is also important to distinguish between genetic and epigenetic determinants of immune memory. MR captures only the genetically determined component of IgG variation and does not reflect epigenetic reprogramming or trained immunity induced by viral exposure or vaccination (Schluter, van Elsas, Priem, Ziogas, and Netea, 2025; Yu, Qiu, and Zhao, 2025). Beyond these layers, the functional quality of IgG is further modulated by post-translational modifications. According to the Paracentral Dogma of glycomedicine proposed by Wei Wang, glycosylation acts as the third alphabet of life, significantly expanding the functional diversity of proteins beyond the genetic template (Wang, 2023). This Sugar Code—the specific glycan structures on the IgG Fc region—serves as a context-dependent molecular signal that switches between pro- and anti-inflammatory functions (Cao, Hu, Li, and Wang, 2025). Such functional heterogeneity may explain the Orthodox vs. Paradox clinical phenotypes observed in vaccine responses, where individuals with similar genetically predicted IgG quantities exhibit divergent outcomes in dermatitis due to variations in their antibody glycoforms. Since MR cannot account for these non‑genetic influences, the findings should not be interpreted as evidence of vaccine‑specific effects. This distinction directly addresses concerns raised in previous observational studies reporting heterogeneous associations between measles‑mumps‑rubella vaccination, measles infection, and eczema‑related outcomes (Gruber et al., 2008; Navaratna et al., 2021; Olesen et al., 2003).

The null findings for the remaining IgG traits are consistent with prior epidemiological evidence suggesting that most childhood vaccinations do not increase the risk of AD or ACD (Ayasse et al., 2021). These results also align with studies indicating that associations between vaccination and dermatitis may be antigen‑specific or context‑dependent rather than universal across immune exposures (Gehrt et al., 2021; Hoffmann, Thiesson, Johansen, and Hviid, 2024). The absence of associations for other IgG traits in our MR analysis suggests that genetically predicted antiviral immune memory does not broadly influence dermatitis risk.

Several limitations should be acknowledged. First, the use of a relaxed SNP selection threshold (P < 5 × 10⁻⁶) was necessary due to the limited number of genome‑wide significant variants available for IgG traits. Although this approach is common in MR studies of immune phenotypes (Long et al., 2023), it may increase the risk of including false‑positive variants. However, the consistency across multiple sensitivity analyses lends support to the robustness of the findings. Second, IgG levels reflect cumulative immune exposure from both vaccination and natural infection, and MR cannot distinguish between these sources. Third, all datasets were derived from individuals of European ancestry, which reduces population stratification but limits generalizability to other populations. Differences in infection history, vaccination coverage, and environmental exposures across populations may also influence IgG distributions. Finally, summary‑level data prevented stratified analyses by age or sex, which may be relevant given known demographic differences in dermatitis prevalence.

In conclusion, this MR study identified a suggestive association between genetically predicted anti‑rubella virus IgG levels and reduced allergic contact dermatitis risk. However, the association did not withstand multiple‑testing correction and should be interpreted as exploratory. No causal effects were observed for other virus‑specific IgG traits. Because IgG reflects immune memory from both vaccination and natural infection, these findings do not indicate vaccine‑specific effects. Further research, including replication studies and mechanistic investigations, is needed to clarify the immunological pathways linking antiviral immune memory with dermatitis susceptibility.

## Ethics approval and consent

Not applicable.

## Funding information

This study was supported by the National TCM inheritance Innovation research project (2023GLYB07) and the National Traditional Chinese Medicine Inheritance and Innovation Center, the First Affiliated Hospital of Guangzhou University of Chinese Medicine. Additionally, it was also supported by the Guangzhou University of Chinese Medicine Teaching Quality and Teaching Reform Project (2025106) and the Guangdong Provincial Department of Education Postgraduate Education Innovation Plan Funding Project (2025ANLK_020).

## CRediT authorship contribution statement

**Zidan Ouyang:** Writing – original draft, Supervision, Software, Project administration, Investigation, Conceptualization. **Jiajian Lin:** Writing – original draft, Visualization, Supervision, Project administration, Investigation, Formal analysis, Conceptualization. **Jing Wu:** Writing – review & editing, Writing – original draft, Validation, Resources, Methodology, Data curation. **Xiaojun Wang:** Writing – original draft, Visualization, Software, Resources, Methodology, Data curation. **Haijiao Wang:** Writing – review & editing, Visualization, Supervision, Resources, Methodology, Formal analysis, Conceptualization. **Li Chen:** Writing – original draft, Visualization, Software, Methodology, Formal analysis, Conceptualization.

## Declaration of competing interest

The authors declare that they have no known competing financial interests or personal relationships that could have appeared to influence the work reported in this paper.

## Data Availability

No data was used for the research described in the article.

## Appendix Supplementary materials

Fig. S1. The funnel and leave-one-out plot between genetically predicted anti‑rubella virus IgG levels and allergic contact dermatitis (ACD). (A) Funnel plot assessing potential horizontal pleiotropy. (B) Leave‑one‑out analysis demonstrating that no single SNP drives the overall MR estimate. Image, image 1 dummy alt text

Image, application 2

Table S1 F-statistical for instrumental variables associated with virus-specific IgG levels. Image, application 3

Table S2 Detailed information for all instrumental variables used in the MR analysis. Image, application 4
