# Supplementary figures and images for: Genetically predicted anti‑rubella virus IgG levels and dermatitis risk: A two‑sample Mendelian randomization study
Source: Virus Res. 2026 Apr 15;367:199726. doi: 10.1016/j.virusres.2026.199726 (PMC13099477; doi:10.1016/j.virusres.2026.199726)

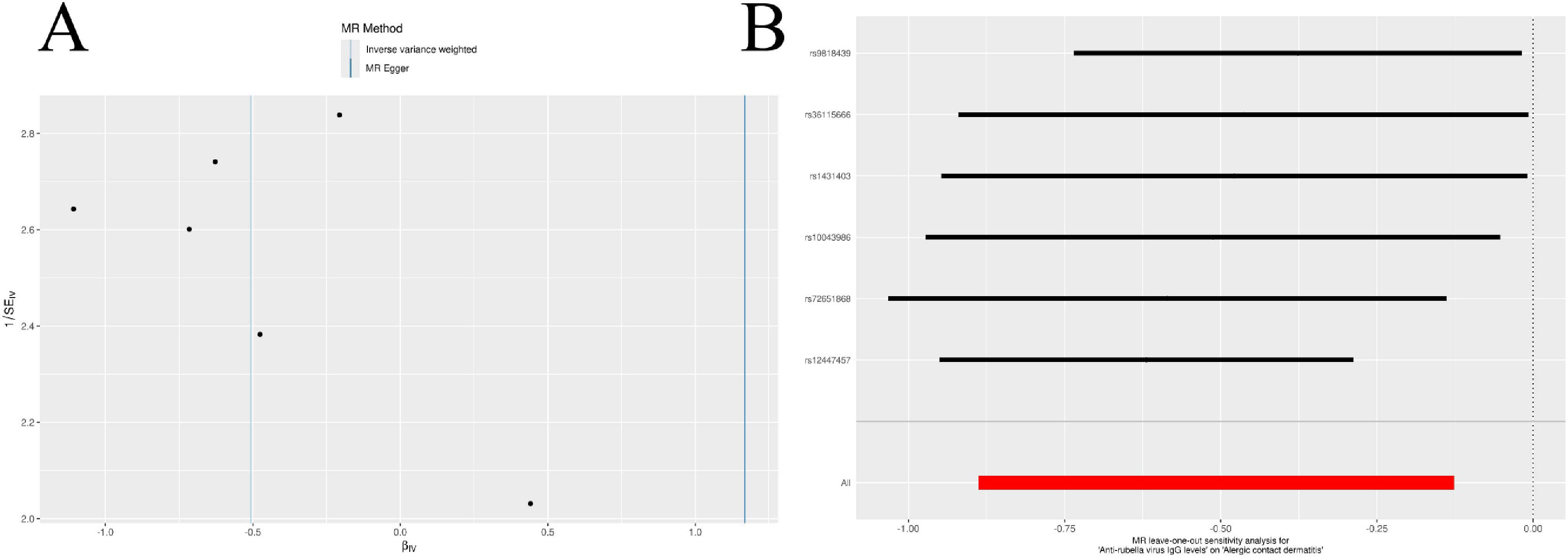

Supplement: Supplementary file 1 — Fig. S1. The funnel and leave-one-out plot between genetically predicted anti‑rubella virus IgG levels and allergic contact dermatitis (ACD). (A) Funnel plot assessing potential horizontal pleiotropy. (B) Leave‑one‑out analysis demonstrating that no single SNP drives the overall MR estimate. [file mmc1.jpg]
